# Immunodeficiency in Children With Invasive Pneumococcal Disease

**DOI:** 10.7759/cureus.98716

**Published:** 2025-12-08

**Authors:** Ahmed M Almesfer, Mohammed M AlSaiary, Fahad M Alharbi, Sami I Alradhi

**Affiliations:** 1 Department of Pediatrics, Pediatric Allergy and Immunology Unit, Maternity and Children Hospital, Dammam, SAU; 2 Department of Pediatrics, Pediatric Infectious Diseases Unit, Maternity and Children Hospital, Dammam, SAU; 3 Department of Pediatrics, Pediatric Gastroenterology Unit, Maternity and Children Hospital, Dammam, SAU; 4 Department of Pediatrics, Pediatric Hematology and Oncology Unit, Maternity and Children Hospital, Dammam, SAU

**Keywords:** antibody deficiency, children, complement pathway, immunodeficiency, invasive pneumococcal disease

## Abstract

Invasive pneumococcal disease (IPD) continues to be a significant contributor to childhood morbidity and mortality. Such occurrences persist despite global vaccination initiatives. Previously unrecognized immunodeficiencies are highlighted due to the presence of IPD in otherwise healthy children. This narrative review has examined the immunologic mechanisms by synthesizing evidence from multicenter studies. These immunologic mechanisms predispose children to IPD. The most common defects of the immune system include antibody and complement deficiencies, congenital asplenia, or innate immune signaling disorders involving *MYD88* and *IRAK4*. Even though pneumococcal conjugate vaccines have played their role in drastically reducing the disease burden, the occurrence of breakthrough infections continues. This happens due to non-vaccine serotypes and impaired immune responses. Early identification of underlying immune defects can be achieved through a structured postinfection evaluation. Recognizing IPD as a potential indicator of immunodeficiency allows clinicians to individualize management. This is achieved through optimization of vaccines, antibiotic prophylaxis, and long-term follow-up. Thereby, the outcome is decreased recurrence and advancing equitable prevention of severe pneumococcal disease in children.

## Introduction and background

Pneumococcal disease (PD) is a leading morbidity among children globally caused by the encapsulated bacterium *Streptococcus pneumoniae*. PD encompasses sinusitis, otitis media, meningitis, bacteremia, sepsis, and pneumonia. The invasion of this bacterium in the sterile sites of the body, such as CSF, blood, peritoneal, or pleural spaces, results in invasive pneumococcal disease (IPD). Although there has been advancement in antimicrobial therapy and vaccination, the threat of IPD remains significant for children worldwide [1]. Before the introduction of pneumococcal conjugate vaccines (PCVs), infections caused by *S. pneumoniae* were the reason behind the deaths of almost one million children each year [1]. Mortality was commonly observed in middle- and low-income countries [1]. The burden of PD declined drastically when PCV7, PCV10, and PCV13 were introduced. In 2002, the incidence of IPD in Australia was noted as 11.5 per 100,000 individuals. However, the incidence decreased between 2016 and 2018 to 7.8 per 100,000 individuals [2-4]. Furthermore, the incidence of IPD in the United States has also declined in children under five years of age. From 1998 to 2018, the cases dropped from 95 to seven per 100,000 individuals [5]. Nonetheless, *S. pneumoniae* still causes 300,000 deaths every year in children under five years of age [5]. This burden is driven by incomplete immunization coverage, non-vaccine serotypes, and underlying factors of host susceptibility, such as immunodeficiency [1].

There are multiple factors that govern the pathogenesis of pneumococcal infections. This depends on the competency of the host immune system and the virulence of the bacterium. Usually, the host remains asymptomatic when bacteria colonize the nasopharynx. However, the bacteria may spread to other organs and systems, such as the lungs, bloodstream, and central nervous system, when mucosal barriers are breached or host immune defenses are impaired [6]. The prevention of invasive diseases is accomplished by the collaborative efforts of multiple immunological components. Opsonizing antibodies against the polysaccharide capsule of the bacterium are generated by humoral immunity. These are essential to be cleared by phagocytosis [7]. Opsonization is enhanced by complement pathways such as alternative, classical, and lectin pathways. The eradication of bacteria is also accomplished through the assembly of the membrane attack complex and the accumulation of complement fragments, such as C3b [8]. Encapsulated organisms are filtered by the splenic macrophages from the bloodstream [9]. The cytokine production is triggered by the receptors of innate immunity, such as Toll-like receptors (TLRs). This coordinates the response of the inflammatory system [10]. If any of these mechanisms, including complement or antibody deficiency, problems in the TLR signaling cascade (such as MYD88 or IRAK4 mutations), or congenital asplenia, are lacking in children, they are at a higher risk of recurrent or severe IPD [11,12].

Invasive pneumococcal infection is becoming increasingly recognized as a possible early sign of immunodeficiency [11]. These immune defects may be primary, resulting from genetic abnormalities, also known as inborn errors of immunity (IEI), or secondary to conditions such as malignancy, immunosuppressive therapy, or human immunodeficiency virus infection. Primary immunodeficiencies commonly affect antibody production, complement function, or innate immune pathways, which is clinically significant because such defects predispose individuals to infections caused by encapsulated bacteria, including *S. pneumoniae*. A French multicenter study by Gaschignard et al. [11] identified primary immunodeficiencies in 10% of children with IPD. They also reported that this proportion rises to 26% in children who are older than two years. These primary immunodeficiencies included antibody deficiencies, complement defects (C2 or C3), congenital asplenia, and signaling defects in MYD88 and IRAK4. Similarly, 40% of children with recurrent IPD were found to be deficient in the complement system by Ingels et al. [12]. The most commonly identified defect was C2 deficiency. Furthermore, Butters et al. [1] reported that immunodeficiency was found to be present in children presenting with their first episode of IPD. The percentage of such children was between 1.3% and 10.5%, and in children with recurrent IPD, this prevalence rose to 66%. The most identified conditions were antibody and complement deficiencies. Butters et al. recommended that all children older than two years with pneumococcal meningitis or complicated pneumonia should undergo immune evaluation, even after a single episode of IPD. More recent prospective studies have reinforced these observations. In the United Kingdom, immune abnormalities were identified in 8% of children with IPD [13]. While in Australia and New Zealand, the abnormality rate was found to be 12%. Phuong et al. [14] strongly supported routine immune evaluation after IPD diagnosis.

A delicate balance between colonization and invasion reflects the interaction between *S. pneumoniae* and the human immune system. In children, prevention of bacterial dissemination relies on the integrated function of mucosal immunity, complement activation, and efficient splenic clearance. However, in individuals with subtle immune irregularities, the otherwise harmless state of nasopharyngeal carriage may transition to invasive bloodstream infection. These immune defects may not be clinically apparent during early childhood. This shift underscores the paramount role of host immune integrity, often outweighing bacterial virulence, in determining disease progression. Consequently, IPD may serve as a clinical marker of impaired innate and adaptive immune responses [12,14]. Susceptibility to PD is influenced by both the transient immaturity of the developing immune system and congenital immunological defects affecting antibody production, pattern-recognition receptor signaling, or complement pathways. Advances in genomic studies have expanded the concept of IEI, demonstrating that single-gene defects involving TLR pathways, NF-κB activation, or cytokine signaling can increase susceptibility to infections with encapsulated organisms [15]. These findings underscore how failure at humoral, cellular, or molecular immune checkpoints can culminate in severe invasive bacterial disease.

Moreover, there has been a significant increase in the importance of the IPD study in otherwise healthy children in the field of modern genomic medicine. IPD now serves as a sentinel model, emphasizing the critical role of well-described immune pathways in host defense. Over the past decade, whole-exome sequencing and next-generation sequencing have revealed multiple monogenic causes of severe bacterial infections. Variants in genes regulating complement activity, antibody maturation, and TLR signaling, including IRAK4, MYD88, NFKB1, and IKBKG, have expanded the recognized spectrum of IEI associated with pneumococcal susceptibility [16,17]. Diagnostic precision is not only enhanced by these discoveries, but it also opens new ways for personalized management. Importantly, unusually severe or recurrent IPD is underscored by this principle. This warrants a comprehensive evaluation even in the absence of overt clinical immunodeficiency. This evaluation should integrate both conventional immune testing and molecular diagnostic approaches [18].

This narrative review was conducted through a comprehensive search of PubMed, Google Scholar, and the Cureus database for studies published between 2015 and 2025. Keywords included “invasive pneumococcal disease”, “children”, “immunodeficiency”, “antibody deficiency”, and “complement pathway”. Preference was given to peer-reviewed clinical studies, meta-analyses, and reviews published in English. Reference lists of relevant papers were also screened to identify additional sources. The objective of this review was to describe the immunologic mechanisms associated with IPD in children and to highlight relevant considerations for clinical evaluation and preventive care.

## Review

Recent years have seen a significant change in the epidemiology of IPD, especially among children. Vaccine rollout played an important role in this evolution. However, antibiotic resistance, the emergence of atypical clinical presentations, and shifts in serotype patterns also facilitated the evolution. Surveillance data from the CDC indicate that there has been a dramatic fall in the rates of IPD in the United States among children under five years of age. This decrease can be attributed to the introduction of conjugate pneumococcal vaccines. Yet, the concern remains the same for the persistent burden of non-vaccine serotypes and evolving host-pathogen dynamics [5]. A systematic review was carried out on serotype distribution across children. The study reported that approximately 28% of pediatric IPD cases are now accounted for by non-PCV13 serotypes (such as 10A, 12F, and 15B/C), especially in countries with established PCV13 programs. This highlights the fact that pneumococcal epidemiology continues to evolve in the vaccine era [19]. Keeping this in view, the influence of host immune factors, including covert immunodeficiencies, becomes increasingly important to consider. The susceptibility to first-time infection is not the only thing affected by host immune factors. The shifting patterns of severity, recurrence, and vaccine breakthrough in pediatric patients are also influenced by the factors of host immunity.

Epidemiology of IPD

The past 20 years have seen a substantial change in the epidemiology of pediatric IPD globally. This resulted largely from the introduction of PCVs and evolving serotype distributions. WHO recognizes PD as a major contributor to child mortality. The estimations have also reported that PD has resulted in the deaths of almost one million children every year in the pre-vaccine era. This mostly occurred in low- and middle-income countries [20]. A dramatic decline in the incidence of IPD in children has been reported by large population-based surveillance studies in high-income settings. For example, a study conducted in Massachusetts (USA) on children less than 18 years of age reported that the incidence of IPD decreased to 72% between 2002 and 2021 following PCV implementation (incidence rate ratio: 0.28). In those aged less than two years, the incidence declined from approximately 33.5 per 100,000 to about 8.6 per 100,000 [21]. A cohort study in England conducted from 2003 to 2019 observed similar trends. The highest incidence was reported in infants aged 0-1 years (15.52 per 100,000 person-years). Following the introduction of PCV7, incidence declined to 3.28 per 100,000, and it further decreased to 1.41 per 100,000 in the late post-PCV13 period [22].

However, the burden of IPD remains substantial despite these declines. This holds true for younger age groups and for settings with lower vaccine coverage or different serotype patterns. A cohort study estimated that between 0.85 and 1.32 million cases of PD in children under five years of age may still occur annually. This happens because of serotypes covered by PCV10, PCV13, PCV15, and PCV20. It has direct medical costs that can be estimated up to USD 136 million [23]. The children younger than two years of age were noted to be at the highest risk of IPD by the European Centre for Disease Prevention and Control. In many countries, children under five years of age account for the majority of serious IPD cases [24]. Moreover, disparities in geography and socioeconomic status are quite noticeable. The incidence of IPD is multiple times higher in low-income regions compared to industrialized countries [25]. Table 1 demonstrates this comparison really well. The incidence of IPD in children under five in sub-Saharan Africa and South Asian countries remains five- to 10-fold higher than in industrialized countries. In some parts of Africa, rates reach up to 60-800 cases per 100,000 child-years. However, in the United States, these rates are 7 per 100,000. These disparities could be the result of differences in vaccine access, nutrition, and healthcare infrastructure.

**Table 1:** Comparative incidence table of IPD in low-income and industrialized countries IPD, invasive pneumococcal disease; PCV, pneumococcal conjugate vaccine

S. no.	Region	Country	Approximate incidence of IPD in children under 5	Reference
1	Low income	Bangladesh	36.3 per 100,000 child-years (0-59 months)	[25]
2		Africa	60-797 per 100,000 in children <2 years in Africa	[20]
3	Industrialized	United States	~7 per 100,000 children <5 years in recent years post-vaccine	[5]
4		Europe	~ 44.4 per 100,000 children <2 years in Europe in the pre-PCV era	[20]

Pathophysiology and host immune mechanisms

There is a delicate interplay between bacterial virulence factors and the competence of the host’s immune system that gives rise to the pathogenicity of *S. pneumoniae*. The nasopharynx in the human body is the first place that these bacteria colonize. This stage remains asymptomatic in most healthy individuals. When the immune defenses fail or host barriers are breached, the disease progresses. The breach of host defenses allows dissemination to normally sterile sites such as the lungs, blood, or CSF. The principal virulence determinant of *S. pneumoniae *is the polysaccharide capsule. It provides resistance against phagocytosis and complement-mediated killing [26]. Tissue invasion and survival are enhanced further by pneumolysin, autolysin, and surface proteins such as PspA. These chemicals interfere with complement activation and epithelial integrity [27]. However, there are multiple layers of host defense mechanisms against pneumococcal infections. The first line of protection is provided by the innate immune system. Mucociliary clearance, antimicrobial peptides, and pattern-recognition receptors such as TLRs are the defense mechanisms of the innate immune system. Intracellular cascades are triggered by the activation of TLR2, TLR4, and TLR9. It is mediated by adaptor molecules, including MyD88 and IRAK4. This leads to NF-κB activation and pro-inflammatory cytokine release (IL-6, TNF-α, and IL-1β). The bacterial clearance is promoted when these signals recruit neutrophils and macrophages to the site of infection [28]. In fact, there is marked bluntness in the inflammatory response in such patients who have inborn errors of TLR signaling, particularly MYD88 or IRAK4 mutations. This results in recurrent or severe invasive pneumococcal infections despite normal antibody levels [16,29].

Innate immunity is represented by another key component known as complement activation. C3b deposition is generated on the bacterial surface by the classical and alternative pathways. This facilitates opsonization and subsequent phagocytosis by neutrophils. The opsonic activity is markedly reduced when regulatory proteins such as Factor I and Factor H or early complement components (C2, C3, and C4) are deficient. There is also a strong relationship between these deficiencies and recurrent IPD [12]. Similarly, encapsulated organisms are filtered by the spleen. Therefore, children with congenital or functional asplenia exhibit severely reduced clearance of pneumococci. Hence, they are at risk of overwhelming post-splenectomy infection [14]. On the other hand, long-term and serotype-specific protection is provided by adaptive immunity through antibody-mediated responses. Pneumococcal polysaccharides are recognized by the activation of B-cells and class-switched IgG2. This enables complement fixation. The same principle is followed by the vaccine-induced immunity. It elicits opsonizing antibodies against common serotypes. However, response failure is observed in children with antibody deficiencies such as X-linked agammaglobulinemia (XLA), common variable immunodeficiency (CVID), or specific polysaccharide antibody deficiency (SPAD). This leaves such children vulnerable even after immunization [13].

The determination of disease outcome has been deliberated by recent studies through the balance between host defenses and the adaptation of bacteria. For example, genomic analyses show that pneumococcal lineages can modulate capsular thickness and pneumolysin expression to evade immune recognition [30]. Alternatively, mucosal immunity and macrophage function are compromised by some host factors such as HIV infection, malnutrition, or concurrent viral illness. This further tips the balance toward invasion [31]. The understanding of these cellular and molecular interactions explains the reason why some children develop invasive disease despite vaccination. It also highlights the importance of screening for immunologic abnormalities when IPD presents atypically or recurrently.

Spectrum of immunodeficiencies linked to IPD

In a subset of otherwise healthy children, IPD may be the first clinical manifestation of an underlying immunologic disorder [11]. There is diversification in the immune abnormalities present in these patients. This indicates toward the fact that multiple pathways control the invasion and passage of *S. pneumoniae* in the host. The defects in the immune system broadly encompass complement activation, antibody production, innate immune signaling, and splenic function, as summarized in Table 2. IPD has been refined after the recognition of these immunologic patterns. From being a purely infectious process, IPD is now a potential indicator of hidden primary or secondary immunodeficiency [11,13].

**Table 2:** Spectrum of immunologic mechanisms linked to IPD in children CVID, common variable immunodeficiency; hyper-IgM, hyper immunoglobulin M syndrome; IKBKG, inhibitor of nuclear factor kappa B kinase subunit gamma; IPD, invasive pneumococcal disease; IRAK, interleukin-1 receptor-associated kinase; MBL, mannose-binding lectin; MYD88, myeloid differentiation primary response 88; NEMO, NF-κB essential modulator; NFKB1, nuclear factor kappa B subunit 1; SPAD, specific polysaccharide antibody deficiency; TLR, Toll-like receptor; XLA, X-linked agammaglobulinemia

Immune mechanism/pathway	Representative defects or conditions	Mechanistic effect	Clinical manifestations/IPD association	Reference(s)
Antibody-mediated immunity	XLA, CVID, SPAD, hyper-IgM syndrome	Defective opsonizing IgG2 and poor antibody response to pneumococcal polysaccharides	Recurrent pneumonia, meningitis, and sepsis; inadequate vaccine response	[1,11]
Complement pathway	Classical (C2, C3) and alternative pathway defects; Factor I, Factor H deficiency	Reduced opsonization and impaired membrane attack complex formation	Recurrent or severe bacteremia, meningitis, poor bacterial clearance	[12,13]
Splenic function	Congenital or functional asplenia (e.g., sickle-cell disease, surgical asplenia)	Loss of splenic filtration and clearance of encapsulated bacteria	Fulminant sepsis or meningitis with rapid progression; high mortality	[11,31]
Innate immune signaling	MYD88, IRAK-4, NEMO (IKBKG), and NFKB1 mutations	Impaired TLR signaling and NF-κB activation, leading to blunted cytokine release	Recurrent meningitis, bacteremia, low CRP, or fever response	[16,27]
Lectin complement pathway	MBL deficiency	Defective recognition and complement deposition on pneumococci	Increased susceptibility to meningitis and pneumonia in infancy	[32,33]
Other genetic/functional modifiers	IRAK1, IRAK4, MYD88 polymorphisms, malnutrition, HIV coinfection	Modulate immune signaling or systemic immune competence	Variable risk for severe or recurrent IPD	[16,34]

The most common category of immunologic defects in children suffering from IPD is constituted by the antibody deficiencies. Gaschignard et al., in their landmark French multicenter study, identified primary antibody deficiencies in nearly half of all children diagnosed with an immune disorder after IPD. The antibody deficiency that they identified includes XLA, hypogammaglobulinemia, and SPAD [11]. Opsonization is impaired by these defects. Additionally, the development of high-affinity antibodies against pneumococcal polysaccharides is also hindered by these defects. This usually results in inadequate vaccine responses. Similar findings were confirmed by Butters et al., who reported that the majority of identified immune defects across more than a dozen international cohorts were antibody-mediated disorders such as CVID and hyper immunoglobulin M syndrome [1]. Total immunoglobulin levels may be normal in children with SPAD but still fail to mount serotype-specific responses. This highlights the importance of both quantitative and functional antibody testing post-IPD evaluation. Furthermore, literature also prominently features complement pathway deficiencies. This particularly involves the classical components C2 and C3. Ingels et al. [12] conducted a Danish national cohort study. They reported that C2 deficiency was observed in 40% of children with recurrent IPD. This underscores the role of classical pathway activation in pneumococcal clearance. Likewise, C2 and Factor I deficiencies were reported by Bijker et al. among children screened after IPD in the United Kingdom [13]. This study put an emphasis on the fact that complement failure puts patients at higher risk of recurrent meningitis and sepsis. When complement-mediated opsonophagocytosis is impaired, bacteria are not effectively marked for destruction, allowing them to persist and multiply in the bloodstream. This explains why these infections can be both severe and recurrent in affected children.

Splenic filtration is another critical host defense mechanism employed against encapsulated organisms. There is always an increased risk of serious pneumococcal sepsis in children who have congenital asplenia or functional hyposplenism. This happens in children who have sickle-cell disease. Butters et al. reported that 1% of children presenting with IPD were asplenic. The mortality in this subgroup approached 50% [1]. This can be verified through the data of an earlier North American study. Schutze et al. [32] reported that 0.8% of pediatric IPD cases occurred in asplenic patients. Half of these patients presented with septic shock. Detection of hyposplenism may be challenging. Therefore, advanced imaging techniques or pitted erythrocyte analysis are recommended for confirmation. The children with inborn errors of innate immunity present a small but clinically significant subset of IPD cases. This is particularly evaluated for the TLR-NF-κB signaling cascade. Downstream cytokine production is impaired by deficiencies in MYD88 or IRAK-4. This also blunts the inflammatory response to pyogenic bacteria. A child from a consanguineous family with MYD88 deficiency was documented by Gaschignard et al. The child presented recurrent pneumococcal meningitis [11]. However, a similar complication was reported by Ingels et al., but it was associated with defective TLR function [12]. Normal antibody and complement profiles are often exhibited by such patients who have signaling defects. However, such patients display poor fever and CRP responses during infection. This results in delayed diagnosis. An increase in susceptibility to pneumococcal and staphylococcal infections has also been found to be linked with broader mutations in IKBKG (NEMO) and NFKB1 [16,28]. Severe outcomes can be prevented through early identification. Early identification allows the use of prophylactic antibiotics, immunoglobulin supplementation, and avoidance of live vaccines.

In addition to all these well-defined immunological defects, pneumococcal susceptibility is also associated with the genetic variants that affect the lectin complement pathway. This particularly encompasses mannose-binding lectin (MBL) deficiency. Genetic studies have been carried out to test this relationship [33,34]. Although the reported results were heterogeneous, complement deposition decreases because of low levels of MBL. This augments the risk of meningitis in infants. In fact, the emergence of IRAK1, IRAK4, and MYD88 gene variation continues as risk modifiers in population-based genetic studies [16,35]. Data from multiple cohorts interpreted together suggest that an identifiable immunologic defect is harbored by 8-15% of children who experience IPD for the first time. However, an identifiable immunologic defect results in recurrent infection in 25% of children [11-13]. The predominant deficiencies include complement and antibody. However, there is some role of splenic abnormalities or rare signaling. These insights reinforce that systematic immune evaluation is required for every episode of IPD in childhood. Recurrence can be prevented by timely recognition through vaccination and prophylaxis. Moreover, early identification also enables family counseling and lifelong follow-up. This transforms the management of IPD from reactive treatment to proactive immunologic care.

Vaccination and breakthrough infections

The global landscape of IPD has been transformed by the PCVs. The outcome of this transformation has been in the form of a significant decrease in child mortality and morbidity. PCV7 was introduced in 2000. This was followed by the availability of PCV10 and PCV13. Following their introduction, vaccine-type diseases declined drastically. The CDC carried out a surveillance in the United States. The outcome of the surveillance study highlighted that IPD caused by PCV7 serotypes in children less than five years of age has reduced by 87% within four years of vaccine rollout [5]. In Europe, the same success was observed after the vaccine rollout. Between 2010 and 2019, a 56% overall reduction in pediatric IPD was achieved by the national immunization program of England. The results were highly prominent in infants less than two years of age [35]. According to the estimates of WHO, conjugate vaccination programs have successfully helped in decreasing child mortality by averting 400,000 deaths annually [36].

Even though there have been many achievements, vaccinated children still face the danger of IPD. Despite receiving age-appropriate vaccination, the cases are represented by these “breakthrough infections.” This is recognized widely as the marker of possible immunologic vulnerability. Multiple factors could be responsible for it. Serotype replacement is the most prominent mechanism. It is the expansion of non-vaccine serotypes filling the ecological niche left by vaccine-targeted strains. The prevalence of serotypes 10A, 12F, 15B/C, and 33F increased following the introduction of PCV13 across Europe and North America [19,37]. A 2023 cohort study showed that non-PCV13 serotypes accounted for nearly one-third of pediatric IPD cases in the vaccine era [37]. Importantly, some “vaccine” serotypes, such as 3 and 19A, have continued to cause disease because of suboptimal immunogenicity or capsular characteristics that hinder antibody-mediated clearance. The next generation of conjugate vaccines, such as PCV15 and PCV20, were developed to broaden coverage and mitigate serotype replacement. Clinical trials indicate that PCV15 elicits non-inferior antibody responses to all PCV13 serotypes and superior responses to additional serotypes 22F and 33F, while PCV20 expands protection to 20 serotypes [38]. Early post-licensure data from the United States show promising immunogenicity profiles and safety comparable to PCV13 [39]. However, the real-world effectiveness of these extended-valency vaccines in immunocompromised children remains under evaluation.

Furthermore, attention has been drawn to host factors by the breakthrough of IPD in individuals who have been fully vaccinated. These host factors are responsible for the impairment of vaccine response, even in the era of higher-valency conjugate vaccines such as PCV15 and PCV20. An underlying immune abnormality was found to be present in 8-12% of children with post-vaccine IPD in the United Kingdom, France, and Australia. As per the studies conducted in these countries, the most common abnormality was either SPAD or complement defect [13,14]. The total immunoglobulin levels may be normal in these children, but the production of adequate serotype-specific IgG may fail to occur following conjugate vaccination. While the clinical significance of SPAD remains controversial, and some reports suggest it does not always necessitate additional interventions, others propose that it may represent a phenotypic manifestation of broader IEI, particularly in the setting of recurrent infections. Therefore, comprehensive immunologic testing should be considered for recurrent or persistent IPD, even when correct vaccination has been carried out. Immunologic testing includes measurement of pneumococcal antibody titers before and after booster doses. If the individuals have HIV infection, functional asplenia, immunosuppressive therapy, or nephrotic syndrome, it can also result in vaccine failure. The reason behind this failure is the bluntness of the antibody response. Such cases warrant revaccination with conjugate followed by polysaccharide vaccine and antibiotic prophylaxis [37]. The Advisory Committee on Immunization Practices recommends additional PCV doses for children with primary immunodeficiency, asplenia, or complement deficiency. Children with these deficiencies are identified to have an elevated risk of recurrent IPD [38]. Understanding the interplay between host immunity and vaccine response is critical to optimizing protection in these vulnerable populations.

The adaptive capability of *S. pneumoniae* has been further illuminated by the recent genomic insights. Emerging serotypes can evade existing vaccine-induced immunity, and this is allowed by the variations in capsular biosynthesis genes and horizontal gene transfer [30]. A dynamic vaccine policy is required due to this continuous evolution. It also necessitates enhanced molecular surveillance and integration of immunogenetic screening for children presenting with breakthrough IPD. Ultimately, vaccination remains the most effective intervention. Yet, despite full immunization, persistent disease underscores the need for vigilant follow-up and recognition of immunodeficiency as a contributing factor.

Diagnostic evaluation and clinical approach

Clinical management and long-term prognosis can be altered substantially after the identification of an underlying immunodeficiency in an IPD. There is a requirement for structured postinfection immune evaluation to differentiate transient immune suppression from persistent or genetic defects because many affected children initially appear healthy. Regardless of age or disease severity, routine screening after any episode of IPD is supported by current scientific evidence. Particularly, if there are cases of meningitis, sepsis, or recurrent infection [1,12]. A stepwise diagnostic framework begins with first-line laboratory investigations readily available in most hospitals. These include a complete blood count with differential to assess leukocyte and lymphocyte numbers, quantitative serum immunoglobulin levels (IgG, IgA, IgM, and IgE), and complement activity measured by CH50 and AH50 assays. Classical pathway deficiency (e.g., C2 or C4) is suggested by low CH50 with preserved AH50. However, a common pathway or C3 defect is present when both values are low. Parallel measurement of CRP and acute-phase reactants helps identify blunted inflammatory responses suggestive of TLR-signaling defects such as MYD88 or IRAK4 deficiency [28,29].

If initial results are normal, a second-tier evaluation should follow, focusing on functional antibody responses. Measurement of pneumococcal IgG titers against multiple serotypes, before and four to six weeks after a conjugate vaccine booster, can identify SPAD, in which baseline immunoglobulin levels are normal but the post-vaccination rise is inadequate. Flow-cytometric analysis of B- and T-cell subsets, including switched-memory B cells and CD4/CD8 ratios, aids in diagnosing combined or cellular defects. Abdominal ultrasonography should be performed in all children with severe or recurrent disease to assess splenic size and function; in equivocal cases, pitted erythrocyte counts or technetium-99m-labelled heat-damaged red-cell scintigraphy can confirm hyposplenism [13]. Genetic testing is warranted when primary investigations reveal abnormalities or in children who present with recurrent IPD, unusual pathogens, or a family history of early-onset sepsis. Next-generation sequencing panels targeting IEI can detect rare defects in MYD88, IRAK4, NFKB1, IKBKG (NEMO), and complement genes. In a multicenter study by Phuong et al., early genomic screening identified IEI in 8% of children with IPD, allowing tailored prophylaxis and family counselling [14]. Genetic results must always be interpreted in the clinical context, as some variants of uncertain significance may not confer functional impairment.

From a clinical management perspective, preventive strategies can be timely initiated when immunodeficiency is recognized early. Conjugate revaccination, regular immunoglobulin-replacement therapy, and long-term antibiotic prophylaxis are beneficial for children who have a confirmed complement or antibody defect. However, the life-saving measures for hyposplenic or asplenic patients include penicillin prophylaxis and prompt treatment of febrile illness [37]. Families should receive education on fever management and emergency access to care. Ongoing monitoring and periodic reassessment of immune parameters can be ensured through coordination with immunology specialists. This is necessary because some antibody deficiencies in early childhood may normalize over time. The differentiation between transient post-infectious hypogammaglobulinemia and genuine primary immunodeficiency should be considered additionally. Studies from Ingels et al. and Bijker et al. report that up to one-third of abnormal immunologic findings may resolve within six months, highlighting the importance of follow-up testing [12,13]. Definitive classification and genetic confirmation should be prompted when serious bacterial infections recur or abnormalities persist beyond a year. A simplified diagnostic algorithm recommended by European and Australasian pediatric societies prioritizes (1) baseline screening after first IPD; (2) extended functional testing after recurrence or meningitis; and (3) genetic analysis for confirmed immune dysfunction or family clustering [13,14]. Implementing this stepwise approach helps clinicians recognize covert immunodeficiency early, prevent further invasive disease, and reduce long-term morbidity.

Knowledge gaps and future directions

Although substantial progress has been made in understanding the immunologic basis of IPD in children, important gaps remain that limit translation into clinical practice. Most available studies are small, single-center, and conducted in high-income countries, leading to underrepresentation of low- and middle-income settings where the burden of IPD remains highest. Global data on the prevalence and distribution of IEI are scarce, and many regions lack access to advanced immunologic or genomic testing [1,14]. Establishing multicenter registries and biorepositories in underresourced areas would help define the true epidemiology and genetic diversity of IEI associated with IPD.

Another unresolved challenge is differentiating transient post-infectious immune suppression from genuine primary immunodeficiency. Prospective longitudinal studies following immune recovery after IPD are limited, yet such work is crucial to avoid overdiagnosis and unnecessary lifelong therapy [12]. Similarly, no universally accepted screening algorithm exists for post-IPD immune evaluation. Current recommendations vary widely among countries and institutions, and their diagnostic yield has never been validated through randomized or cost-effectiveness studies [13].

Moreover, the molecular mechanisms of polygenic risk and epigenetic regulation in pneumococcal susceptibility are still poorly characterized. These mechanisms can be elucidated with the help of emerging technologies. Technologies such as whole-exome sequencing, single-cell transcriptomics, and host-pathogen interaction modelling could help with the refinement of patient stratification. Finally, focus should also be put on vaccine research. The evolution of vaccines toward pan-serotype or protein-based formulations can provide broader, immunity-driven protection across populations with variable immune competence [30]. Addressing these knowledge gaps will bridge the divide between immunologic discovery and equitable, evidence-based prevention of childhood IPD worldwide.

## Conclusions

IPD in children represents the crossroad between infection and immunology. The underlying immune vulnerabilities are highlighted by the presence of recurrent or severe IPD in apparently healthy children. Although the global disease burden has drastically reduced after the introduction of PCVs, IPD continues its manifestation. The reason is an identifiable immunodeficiency present in almost one-quarter of children affected by IPD. The most common defects include antibody and complement deficiencies, congenital asplenia, or innate immune signaling disorders involving MYD88 and IRAK4. The unique opportunity to have an early diagnosis and preventive steps through targeted vaccination, antibiotic prophylaxis, and family counseling is therefore provided by the routine post-IPD immune evaluations. The combination of basic hematologic, complement, and humoral screening with functional antibody testing and genetic analysis in the stepwise investigation offers a clinically meaningful and cost-effective approach. In the future, the focus should be on the expansion of genomic access in low-resource settings, developing broader-spectrum vaccines capable of overcoming serotype replacement, and harmonizing diagnostic algorithms. There is a need to recognize IPD not only as an infectious event, but it also needs to be accepted as a potential sentinel of immunologic disease. The outcome can be transformed through the prevention of recurrence, improved survival, and lifelong health optimization for children worldwide.
